# Human Rabies — Texas, 2021

**DOI:** 10.15585/mmwr.mm7149a2

**Published:** 2022-12-09

**Authors:** Dawn Blackburn, Faisal S. Minhaj, Roukaya Al Hammoud, Lillian Orciari, Jael Miller, Trevor Maness, Jon Stewart, Brittany Singletary, Elvia Ledezma, Misti Ellsworth, Andrea Carlo-Angleró, Michael Niezgoda, Crystal M. Gigante, Agam K. Rao, Panayampalli S. Satheshkumar, Gloria P. Heresi, Amanda Kieffer, Ryan M. Wallace

**Affiliations:** ^1^Texas Department of State Health Services Region 6/5 South, Houston, Texas; ^2^Division of High Consequence Pathogens and Pathology, National Center for Emerging and Zoonotic Diseases, CDC; ^3^Epidemic Intelligence Service, CDC; ^4^Division of Infectious Diseases, Department of Pediatrics, McGovern Medical School at UTHealth Houston and Children's Memorial Hermann Hospital, Houston, Texas; ^5^Texas Department of State Health Services Region 8, San Antonio, Texas.

In late August 2021, a boy aged 7 years was bitten by a bat while he was playing outside his apartment home in Medina County, Texas. He informed his parents; however, no rabies postexposure prophylaxis (PEP) was sought because there were no visible bite marks, and the family was unaware that contact with a bat, including in the absence of visible bite marks, might cause rabies. Approximately 2 months later, the child was hospitalized for altered mental status, seizures, and hypersalivation and ultimately received a diagnosis of rabies. Experimental therapies were attempted; however, the child died 22 days after symptom onset. Fifty-seven persons who met criteria for suspected or known exposure to infectious secretions in this case were advised to consult with a medical provider about the need for rabies PEP in accordance with Advisory Committee on Immunization Practices (ACIP) guidelines (*1*). Rabies, an acute, progressive neuroencephalitis, is nearly always fatal. Although dogs are the most common source of human rabies deaths worldwide and account for an estimated 59,000 annual cases of human rabies globally (*2*), bats are the most common source of domestically acquired rabies in the United States and have been implicated in 31 (81.6%) of 38 human infections since 2000 (*3*). Attempts to prevent death or poor neurologic outcomes once rabies symptoms develop have been largely unsuccessful (*4*). Administration of rabies PEP, comprising rabies immunoglobulin and a series of doses of rabies vaccine, is critical to preventing rabies after an exposure; enhanced public education about the risk posed by bats, and the availability of PEP to prevent rabies, is needed.

On October 21, 2021, the boy aged 7 years was evaluated at a freestanding emergency department (facility A) for a 2-day history of right-hand pruritus and right upper extremity pain. He was given an oral steroid and discharged home. The following day, he was assessed at a different hospital emergency department (facility B) for a rash on the right side of his head, right scapular area, and right hand and arm along with continued pain in his right arm. He received a diagnosis of presumptive herpes zoster (shingles) and was prescribed a 5-day course of acyclovir along with antihistamines and ibuprofen. One day later, on October 23, he returned to facility B with delusions and worsening pruritus of his forehead and was discharged with diazepam for spasms and gabapentin for pain. Later that same day, he returned to facility B with nausea, vomiting, fever of 104°F (40°C), hypersalivation, and change in mental status, including confusion and delusions; he was intubated for airway protection. That evening, he was transferred to facility C, where he was admitted and began treatment with empiric antimicrobial drugs for presumed central nervous system infection. Initial testing included cerebrospinal fluid (CSF) and blood cultures and testing for herpes simplex virus, varicella zoster virus, enterovirus, mycoplasma, Bartonella, Epstein-Barr virus, and cytomegalovirus; all tests later had negative results. On October 25 (the third day of hospitalization), a diagnosis of rabies was suspected after infectious disease clinicians solicited a detailed history that disclosed the bat bite approximately 2 months earlier. Although the child had reported the bite to parents, no bite marks were seen, and the risk of rabies from bat contact was not considered; therefore, care was not sought. Aggressive intensive care management was initiated in facility C, and the patient began treatment with experimental intrathecal human rabies immune globulin on hospital day 7; however, this regimen was not successful, and the patient died on hospital day 16.

## Public Health Investigation

Once rabies was suspected, saliva, nuchal skin biopsy, serum, and CSF were collected and sent to CDC’s National Rabies Reference Laboratory. On October 27, nuchal skin biopsy and saliva specimens confirmed the presence of rabies viral RNA via real-time reverse transcription–polymerase chain reaction testing, confirming rabies virus infection (*5**,**6*). Sequencing of viral RNA collected from the patient was consistent with rabies virus found in the Mexican free-tailed bat (*Tadarida brasiliensis*), the most commonly reported rabid animal in Texas (*7*). This activity was reviewed by CDC and was conducted consistent with applicable federal law and CDC policy.^†^

Texas Department of State Health Services (DSHS) interviewed family and community contacts to determine potential exposures to the patient during the infectious period, estimated to have commenced on October 5 (2 weeks before symptom onset) (*8*). Once the diagnosis was confirmed, persons who met exposure criteria (i.e., suspected or known exposure to infectious secretions) were advised to speak with a medical provider about administration of rabies PEP in accordance with ACIP guidelines (*1*). Among 10 of the patient’s family members assessed, six met exposure criteria and received PEP (Table). One additional family member elected to receive PEP despite having no reported exposure risk.

**TABLE:** Health care, community, and family contacts* of a human rabies case who met exposure criteria**^†^** and who sought rabies postexposure prophylaxis — Texas, 2021

Contact characteristic	Contact setting, no. (%)			
	Health care	Community	Family	Total
	n = 118	n = 49	n = 10	N = 177
Met exposure criteria	5 (4.2)	46 (93.9)	6 (60.0)	**57 (32.2)**
Sought PEP	1 (0.9)	34 (69.4)	7^§^ (70.0)	**42 (23.7)**

**Abbreviation**: PEP = postexposure prophylaxis.

* Persons having any contact with patient during infectious period.

^†^ Any suspected or known exposure to infectious secretions; contacts meeting exposure criteria were advised to speak with a medical provider about rabies PEP.

^§^ One family member elected to receive PEP despite not having met exposure criteria.

The child had attended school and an extracurricular program during his infectious period. DSHS met with school administration and the extracurricular program director to identify persons who could have met exposure criteria (e.g., sharing of food or exertional face-to-face interactions). Among 49 community contacts, 46 met exposure criteria, and 34 contacts sought PEP. Most who sought PEP were students participating in the extracurricular program because they reported close contact during which tears and saliva were potentially exchanged. Local hospitals and physicians were advised of the potential increased demand for PEP.

Infection preventionists at facilities A, B, and C were provided a health care worker rabies risk exposure assessment tool^§^ that included information about each health care worker’s rabies vaccination status, the amount of time spent with and nature of physical contact with the patient (e.g., kissing or being bitten), and whether there was any contact with the patient’s body fluids while not wearing personal protective equipment. The schedule for rabies PEP was also provided to infection preventionists. Five health care contacts among 118 assessed for exposure risk met exposure criteria; one sought PEP.

Following confirmation that the patient’s exposure was caused by a bat bite outside his residence, DSHS contacted the apartment complex where he had resided and sent email and printed rabies advisories^¶^ to the residents notifying them of the rabies risk from bats and the availability of treatment for exposed persons. Receipt of the health advisory was confirmed by telephone; among 175 residents, 124 (71%) were successfully contacted. Twenty-four residents reported sightings of bats in or around the complex; none reported physical contact with a bat. Evaluation of interviews from residents who reported bat sightings enabled DSHS and local animal control to identify the bat colony location. Immediate remediation of the colony was advised by DSHS and successfully completed by a pest management company.

After the patient’s death, the funeral home and embalmer were contacted to ascertain the possibility of any further potential rabies exposures. No recommendations for rabies PEP were made because appropriate precautions had been taken (*9*).

Among the 42 contacts who initiated PEP, all were determined by DSHS to have completed PEP. No additional human rabies exposures or cases have been identified as a result of contact with this patient or the index bat in the apartment complex.

On October 29, 2021, DSHS issued a news release reporting the case and informing the public that at-risk contacts had been identified and were being assessed regarding the need for rabies PEP. General recommendations were provided for preventing rabies such as not approaching wild animals, seeking medical attention after an animal bite or scratch, and ensuring domestic dogs and cats are up to date with rabies vaccination.

## Discussion

Bats are a reservoir species for rabies virus in all U.S. states except Hawaii. Bat-mediated human rabies deaths increased in 2021 following 2 years with no confirmed cases (*10*). In this case, bite marks were not recognized by the patient’s immediate family members, and there was a lack of awareness of the risk for rabies from a bat in the absence of a visible bite mark, resulting in their not seeking medical care as well as a delay in eliciting the exposure history across multiple health systems. Contact with bats, including bites, is typically recognized by the recipient because of the bite force impact, despite many North American bat species typically having small teeth. Bites might not leave observable puncture marks, and given the high risk for rabies virus transmission from bats, PEP is recommended for any bat contact when a bite or scratch cannot be ruled out. Increased public health outreach and education about the rabies risk associated with bats and that rabies is preventable with PEP is needed. As part of its educational effort, Texas DSHS sponsors an annual rabies poster contest for school-aged children.**

Humans shed rabies virus during the clinical phase of disease; however, there has been no confirmed human-to-human transmission of rabies apart from that occurring through organ or tissue transplantation, including in health care settings. Rabies virus is transmitted through direct contact (such as through broken skin or mucous membranes in the eyes, nose, or mouth) with saliva, tears, respiratory secretions, and brain or nervous system tissue. Use of standard precautions^††^ protects health care workers against potential exposure to rabies. In this investigation, compliance with standard COVID-19 precautions^§§^ at health facilities enabled nearly all health care providers to confidently rule out exposure. However, a significant number of community members received PEP because of possible exposure during the patient’s social activities and lack of reliable information about nature of exposures to the patient from his peers, who were mostly children aged <10 years.

This case serves as a reminder that rabies virus is still present in the United States and that exposures to bats and other mammalian wildlife should always prompt a consultation with public health officials or medical providers. It is important to inform animal control or local public health officials when bats build roosts within and around human dwellings. PEP is highly effective and should be administered as soon as possible after an exposure to prevent rabies.

**Summary**

**What is already known about this topic?**

U.S. human rabies deaths typically result from contact with rabid bats. Rabies is preventable when postexposure prophylaxis (PEP) is promptly administered; once clinical signs develop, the disease is nearly always fatal.

**What is added by this report?**

A young boy was bitten by a bat; multiple persons knew of the exposure but did not recognize the rabies risk in the absence of a visible bite mark. Medical care was not sought until the child developed signs and symptoms 2 months later. One third of the child’s contacts met exposure criteria, and one quarter sought PEP; no secondary cases were detected.

**What are the implications for public health practices?**

Enhanced public education about the risk for rabies associated with bat contact and the importance of seeking PEP if contact occurs is needed.

## Footnotes

* These authors contributed equally to this report.

† 45 C.F.R. part 46, 21 C.F.R. part 56; 42 U.S.C. Sect. 241(d); 5 U.S.C. Sect. 552a; 44 U.S.C. Sect. 3501 et seq.

§ The Rabies Risk Assessment Tool is available by emailing rabies@cdc.gov.

¶ Rabies advisories are available by emailing rabies@cdc.gov.

**
https://www.dshs.texas.gov/idcu/disease/rabies/information/contest.aspx

††
https://www.cdc.gov/oralhealth/infectioncontrol/summary-infection-prevention-practices/standard-precautions.html

§§
https://www.cdc.gov/coronavirus/2019-ncov/hcp/infection-control-recommendations.html
